# Integrative multi-omics analyses reveal transcriptional, metabolic, and hormonal regulation of vernalization-induced flowering in Wucai (*Brassica rapa*)

**DOI:** 10.1186/s43897-026-00246-7

**Published:** 2026-10-10

**Authors:** Yunfei Tian, Danni Yang, Na Liao, Kang Wang, Yuxin Yang, Xinghao Zhou, Ting Li, Wenjie Wang, Xiaoyan Tang, Chenggang Wang, Lingyun Yuan, Guohu Chen

**Affiliations:** 1 Anhui Provincial Engineering Research Center of Horticultural Crop Breeding, College of Horticulture, Anhui Agricultural University, Hefei, 230036 China; 2 Wanxinan Comprehensive Experiment Stations of the Research Institute for New Rural Development, AAU, Anqing, Anhui 246121 China; 3 Yingshang Shili Ecological Agriculture Technology Co., Ltd., Fuyang, 236200 China

**Keywords:** Amino acids, Hormones, Sucrose, Transcription factors, Vernalization

## Abstract

Vernalization, a low-temperature-induced transition from vegetative to reproductive growth, is essential for flowering in many *Brassica* crops, including Wucai (*Brassica rapa* ssp. *chinensis* var. *rosularis*). To elucidate the molecular basis of vernalization in Wucai, we performed an integrative multi-omics analysis of shoot apices from cold-treated plants. Twenty-eight vernalization pathway-related genes, including homologs of *FLC*, *FLM*, *VIN3*, *VRN*, *SOC1*, *SVP*, and *VIL2*, exhibited conserved cold-responsive expression patterns. Transcription factors from the MYB, bHLH, bZIP, and NAC families showed dynamic regulation, implicating key members such as *MYB57*, *bHLH3*, and *ABF4* in flowering control and hormonal signaling. Metabolomic profiling revealed significant enrichment of carbohydrate and amino acid metabolism, with increased levels of sucrose, glucose, galactose, glycine, serine, proline, and arginine, suggesting enhanced mobilization of energy and nitrogen during vernalization. Proline accumulation, together with upregulation of *P5CS*, indicates a potential role in floral transition. Hormonal analysis showed elevated auxin, kinetin, jasmonic acids, and brassinosteroids, with cold-induced *BcDET2* expression correlating with increased brassinosteroid levels and early flowering. Exogenous brassinolide further promoted flowering by activating *FT* and *SOC1* while repressing *FLC*. These findings provide a comprehensive molecular framework for vernalization-regulated flowering in Wucai.

**Supplementary Information:**

The online version contains supplementary material available at https://doi.org/10.1186/s43897-026-00246-7.

## Core

Integrative multi-omics analysis elucidated the molecular mechanisms controlling vernalization-induced flowering in Wucai, revealing a coordinated network of transcriptional regulation, metabolic reprogramming, and hormonal signaling.

## Gene and accession numbers

Sequence data from this article can be found in the BRAD database under the accession number: *MYB57* (BraA01g003740.3C), *MYBH* (BraA01g019220.3C), *bHLH3* (BraA01g020360.3C), *bZIP59* (BraA04g022790.3C), *B3* (BraA05g019760.3C), *ABF4* (BraA05g027980.3C), *HUA1* (BraA05g034410.3C), *KHZ1* (BraA05g034780.3C), *NF-YC9* (BraA06g005970.3C), *RNJ* (BraA06g026930.3C), *TCP7* (BraA06g030710.3C), *WHY3* (BraA06g039670.3C), *CAMTA* (BraA07g031410.3C), *B3* (BraA08g003760.3C), *OCP3* (BraA10g026870.3C), *CAMTA1* (BraA10g031180.3C), *CAD7* (BraA01g001080.3C), *FBR1* (BraA08g020010.3C), *HCT* (BraA01g020780.3C), *ATS3* (BraA10g019590.3C), *AAO* (BraA02g043580.3C), *PS2* (BraA02g021420.3C), *PGM1* (BraA08g032410.3C), *CATc* (BraA08g015510.3C), *HXK1* (BraA09g012100.3C), *ASP1* (BraA05g013640.3C), *CATa* (BraA01g003280.3C), *CATb* (BraA03g059190.3C), *MDH2* (BraA02g003100.3C), *PRKa* (BraA08g008980.3C), *PRKb* (BraA09g033090.3C), *GGAT1* (BraA09g039860.3C), *P5CS* (BraA05g006260.3C), *PGDH3* (BraA03g038870.3C), *SHM1a* (BraA01g001110.3C), *AK3* (BraA02g013600.3C), *SHM1b* (BraA08g021770.3C), *ABCG7* (BraA02g034580.3C), *ABCC2* (BraA05g010420.3C), *ABCG13* (BraA06g004620.3C), *ABCC1* (BraA08g023310.3C), *ABCF22* (BraA10g029220.3C), *ABCG12* (BraA05g018270.3C), *ABCC4* (BraA06g002640.3C), *ABCB14* (BraA09g036730.3C), *ABCG35* (BraA06g011250.3C), *FLC2* (BraA02g003340.3C), *FT* (BraA02g016700.3C), *DET2* (BraA10g023600.3C), *SOC1* (BraA04g031640.3C), *FLC3* (BraA03g004170.3C), *FLC1* (BraA10g027720.3C), *VIN3a* (BraA02g012310.3C), *VIN3b* (BraA03g012460.3C), *VIN3c* (BraA06g040160.3C), *SVP* (BraA09g054750.3C), *VRN1c* (BraA05g028310.3C), *VRN1b* (BraA03g038610.3C), *VRN1a* (BraA01g033970.3C), *FLM1* (BraA06g028520.3C), *FLM2 (*BraA06g028530.3C).

## Introduction

Flowering transition, a pivotal event in plant development, is precisely regulated by a dynamic interplay of genetic networks, endogenous cues, and environmental signals (Bloomer and Dean 2017; Chu et al. 2024). In *Arabidopsis thaliana*, this process is governed by multiple regulatory pathways, including vernalization, photoperiod, gibberellin (GA), autonomous, ambient temperature, and aging pathways, each responding to distinct stimuli and converge on a common set of integrator genes (Freytes et al. 2021; Shen et al. 2022). Key integrators such as *FLOWERING LOCUS T* (*FT*) and *SUPPRESSOR OF OVEREXPRESSION OF CO 1* (*SOC1*) activate meristem identity genes (*LEAFY*, *FRUITFULL*, *APETALA1*) to initiate floral development under favorable conditions (Huang et al. 2024; Qiu et al. 2024). Among these, *FT*, evolutionarily conserved across species, is expressed in leaves and transported to the shoot apical meristem (SAM), and triggers floral initiation (Liu et al. 2021). Furthermore, abiotic and biotic stresses can modulate flowering time through phytohormones and metabolic signaling, including auxin, cytokinin, jasmonate, abscisic acid, ethylene, and brassinosteroids, thereby either accelerating or delaying the reproductive transition to optimize the balance between survival and reproduction (Li et al. 2024; Patra et al. 2024).

Vernalization, the process by which prolonged exposure to cold temperatures promotes flowering, constitutes a key adaptive mechanism in many temperate plant species (Huang et al. 2024; Wu et al. 2024). In *A. thaliana*, the SAM serves as a primary site for cold sensing, where the MADS-box transcription factor *FLOWERING LOCUS C* (*FLC*) acts as a central floral repressor (Lee et al. 2015). Prior to vernalization, the FRIGIDA (FRI) protein maintains high *FLC* expression, which in turn suppresses flowering integrators like *FT* and *SOC1*, as well as *FLOWERING LOCUS D* (*FD*) (Shen et al. 2022; Shi et al. 2023; Zhang et al. 2023b). During cold exposure, *FLC* is epigenetically silenced through histone modifications mediated by the Polycomb Repressive Complex 2 (PRC2). This process is initiated by the cold-inducible protein VERNALIZATION INSENSITIVE3 (VIN3) and stabilized by VERNALIZATION RESPONSE 1/2 (VRN1/VRN2) (Niu et al. 2024), resulting in mitotically stable repression that permits flowering upon returning to warmer temperatures (Salomé, 2022).

Although the core genetic regulators of flowering are highly conserved across plants, recent genomic studies in *Brassica rapa* have revealed increased regulatory complexity resulting from whole-genome triplication (WGT) (Wu et al. 2024; Zhang et al. 2022). The *B. rapa* genome harbors more than 500 flowering-associated genes—nearly twice the number identified in *Arabidopsis*—including multiple paralogs of key regulators such as *FLC*, *FT*, and *CONSTANS* (*CO*) (Wu et al. 2024). For instance, four *BrFLC* homologs (*BrFLC1*, *BrFLC2*, *BrFLC3*, and *BrFLC5*) exhibit differential responses to cold-induced repression, while five *FLOWERING LOCUS M* (*FLM*)/*MADS AFFECTING FLOWERING 1* (*MAF1*) genes (*BrMAF1*–*BrMAF5*) display subfunctionalized expression profiles, collectively modulating vernalization responses across tissues (Akter et al. 2023; Li et al. 2019, 2018). Despite these insights from model species, the molecular basis of flowering regulation remains poorly characterized in economically important subspecies such as non-heading Chinese cabbage (Hong et al. 2021a; Liu et al. 2025).

Wucai (*Brassica campestris* L. syn. *B. rapa* L. ssp. *chinensis* var. *rosularis* Tsen) is a nutrient-rich leafy vegetable, valued for its rich dietary fiber and vitamin content, yet it faces significant agricultural challenges in its native Yangtze-Huaihe Basin (Chen et al. 2019; Liu et al. 2025). As an autumn- and winter-sown crop, vernalization-induced premature bolting reduces both yield and commercial quality. Current breeding strategies aimed at mitigating early bolting are constrained by a limited understanding of the Wucai-specific vernalization pathways. While paralogous genes are presumed to retain functions analogous to *Arabidopsis* orthologs, their potential functional redundancy and divergence in Wucai remain unresolved. Elucidating these mechanisms is crucial for developing late-bolting cultivars while maintaining floral competence. In this study, we employed an integrated multi-omics approach to investigate the molecular basis of the vernalization response in Wucai, aiming to provide novel insights into the evolution of flowering regulation in *Brassica* species and to facilitate targeted improvement of bolting traits.

## Results

### Transcriptome and proteome profiling

**Fig. 1:**
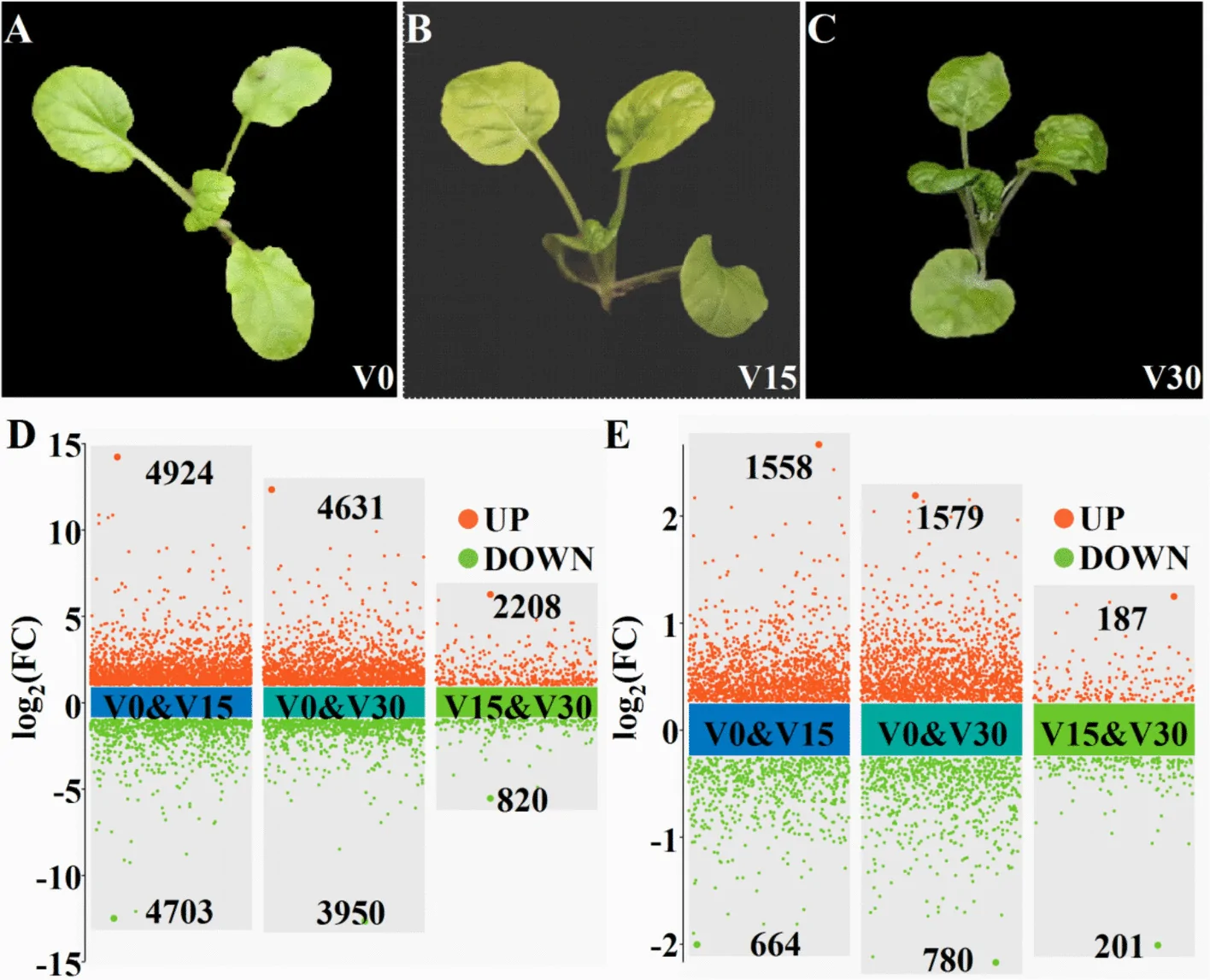
Phenotypic and molecular profiling of Wucai during vernalization. **A-C** Phenotype of Wucai seedlings subjected to 0 (**A**), 15 (**B**), and 30 (**C**) days of vernalization. **D**, **E** Volcano plots depicting DEGs (**D**) and DEPs (**E**); significantly upregulated and downregulated DEGs/DEPs are highlighted in red and green, respectively; the numbers above/below each comparison group indicate the count of up-regulated/down-regulated genes/proteins; the maximum points in different comparison groups represent the extreme value. V0, 0 day vernalization; V15, 15 days vernalization; V30, 30 days vernalization; same below

To investigate the molecular mechanisms of vernalization in Wucai, transcriptomic and proteomic analyses were performed on shoot apices collected at different time points (V0, V15, and V30) during cold treatment (Fig. 1A-C). RNA-sequencing generated approximately 152.62 Gb of clean data from 510 million reads. The overall sequencing quality was confirmed by mean Q20 and Q30 scores of 97.93% and 94.10%, respectively (Fig. S1A-S1B). The clean reads were aligned to the *Brassica rapa* reference genome (V3.0) with a mapping efficiency exceeding 83.53% (Fig. S1C), resulting in the identification of 23,987 annotated genes and 833 novel genes. Proteomic analysis identified 45,573 peptides from 362,608 spectra (Fig. S1D), corresponding to 7,959 annotated proteins and 107 novel proteins (*p* < 0.05). The dataset demonstrated high reproducibility, supported by strong Pearson correlation coefficients among biological replicates (Fig. S2). Furthermore, integrative correlation analysis between transcript and protein levels identified more than 7,700 mRNA-protein pairs, with coefficients of *r* = 0.27, 0.21, and 0.11 for the V0&V15, V0&V30, and V15&V30 comparisons, respectively (Fig. S3). These relatively low correlation values may be attributed to multilayered regulatory mechanisms including non-coding RNA regulation and post-translational modifications.

### Identification of DEGs and DEPs

Comparative analysis identified 12,188 differentially expressed genes (DEGs) and 3,108 differentially expressed proteins (DEPs) across the three comparison groups (V0&V15, V15&V30, and V0&V30). Specifically, 9,627 (4,924 up- and 4,703 down-regulated), 8,581 (4,631 up- and 3,950 down-regulated), and 3,028 DEGs (2,208 up- and 820 down-regulated) were detected in the respective comparisons. Similarly, 2,222 (1,558 up- and 664 down-regulated), 2,359 (1,579 up- and 780 down-regulated), and 388 DEPs (187 up- and 201 down-regulated) were identified (Fig. 1D-E).

**Fig. 2:**
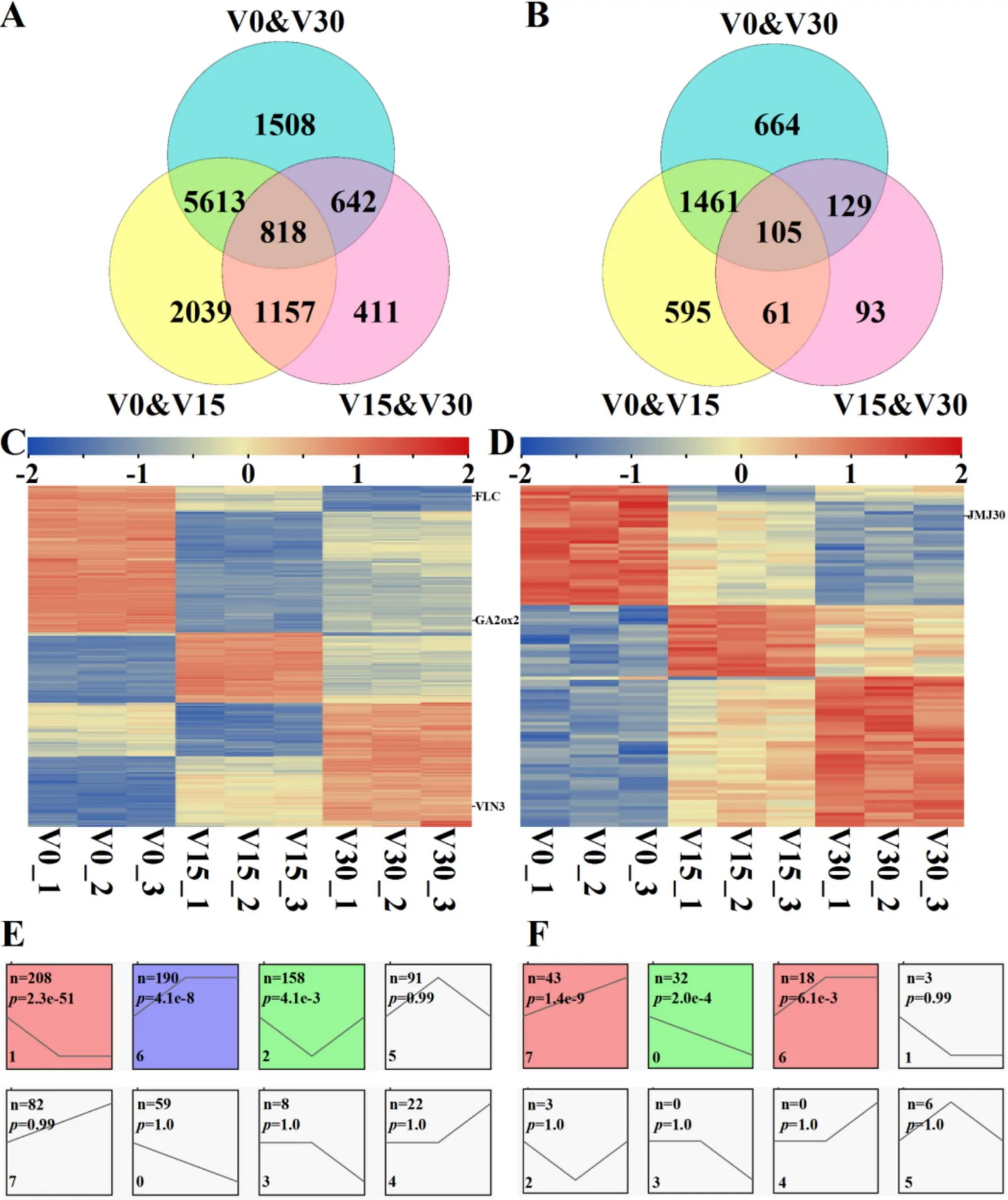
Comparative analyses of DEGs and DEPs. **A**, **B** Venn diagrams of DEGs (**A**) and DEPs (**B**) across the three comparison groups. **C**, **D** Heatmaps of FPKM values for 818 shared DEGs (**C**) and counts of 105 shared DEPs (**D**); row scales were normalized and the values were scaled by log2; red and blue indicate high and low abundance, respectively. **E**, **F** Expression trends of 818 shared DEGs (**E**) and 105 shared DEPs (**F**); profiles 1, 6, 2 for DEGs and profiles 7, 0, 6 for DEPs were significantly enriched (*p* < 0.05)

A total of 818 DEGs and 105 DEPs were shared among all three comparison groups (Fig. 2A-B; Table S2). Heatmap visualization illustrated the dynamic expression patterns of these shared DEGs and DEPs (Fig. 2C-D). For instance, the transcript level of *BraA08g023600.3C* (*GA2ox2*) increased, whereas the level of *BraA03g012460.3C* (*VIN3*) decreased after 15 days of vernalization. The expression of the central floral repressor *FLC* (*BraA03g004170.3C*) progressively decreased with prolonged cold exposure. At the protein level, many DEPs exhibited consistent trends of accumulation or reduction across the vernalization stages. Expression profile clustering further revealed that transcript clusters 1, 6, and 2, together with protein clusters 7, 0, and 6 were significantly enriched among the shared DEGs and DEPs (*p* < 0.05; Fig. 2E-F).

### Identification of vernalization-related genes and transcription factors

**Fig. 3:**
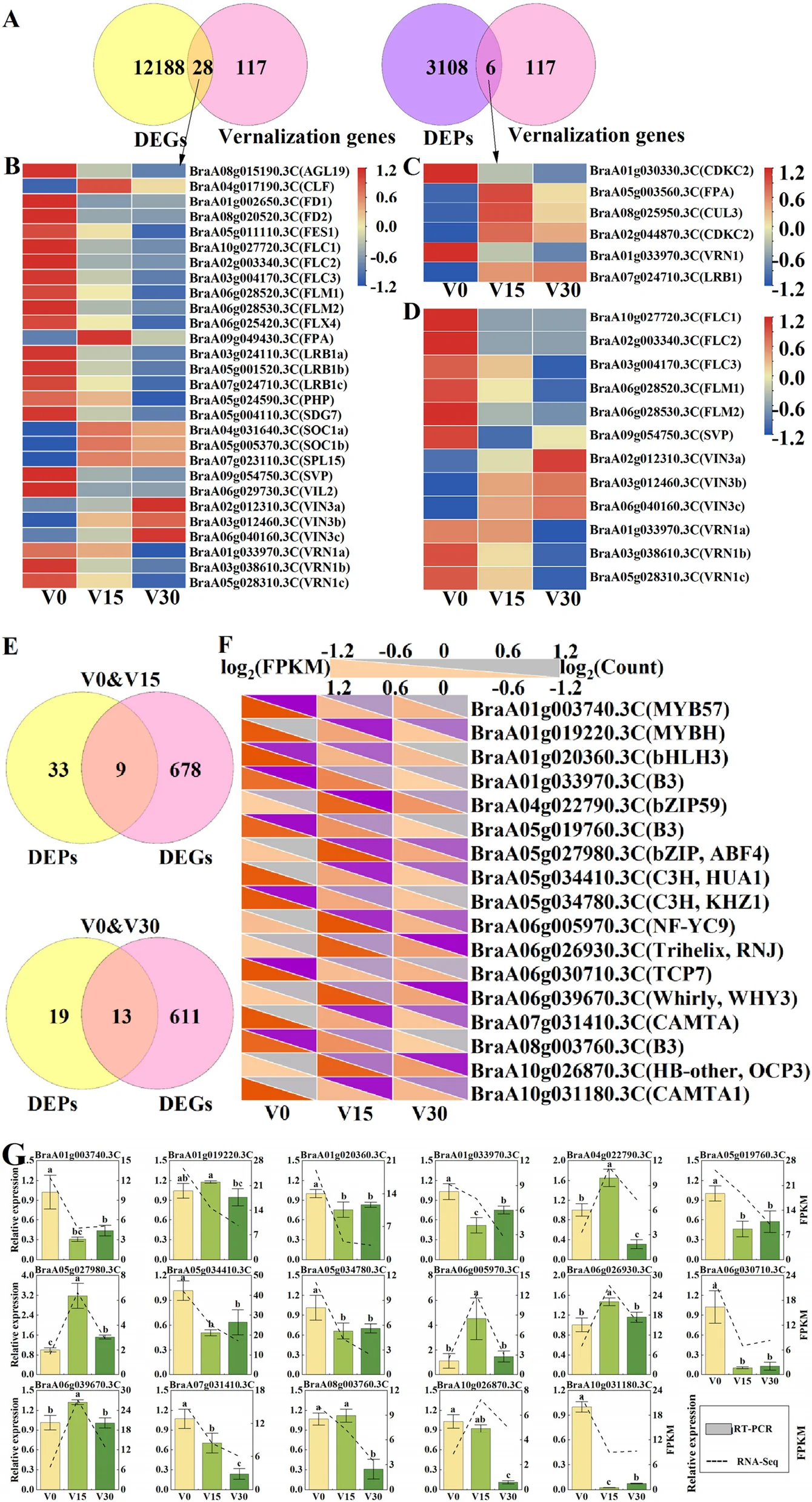
Vernalization-related genes and transcription factors (TFs). **A** Number of DEGs/DEPs in the vernalization pathway. **B**, **C** Heatmaps of DEGs (**B**) and DEPs (**C**) in the vernalization pathway. **D** Heatmap of vernalization genes validated by qRT-PCR. **E** TF counts among DEGs/DEPs in V0&V15 and V0&V30. **F** TF expression trends based on transcriptomic (down) and proteomic (up) data; **G** qRT-PCR (bars, left-axis) and RNA-seq (line, right-axis) of selected TFs; error bars indicate the means ± SEs; lowercase letters indicate significant differences between stages (Duncan’s test, *p* < 0.05). Heatmap row scales were normalized and the values were scaled by log2; heatmap colors indicate FPKM (mRNA) or count (protein), darker colors indicate higher expression levels

Among the 543 flowering-related genes annotated in the *B. rapa* genome (version 3.0), 117 were identified as associated with the vernalization pathway (data not shown). Among these, 28 DEGs and 6 DEPs were identified (Fig. 3A). Key flowering regulators like *FLC*s, *SVP1*, and *VRN1*s were significantly downregulated, while *VIN* 3 s were upregulated during vernalization (Fig. 3B-D).

To identify vernalization-responsive transcription factors (TFs), we analyzed their expression dynamics. A total of 687, 293, and 624 TFs were differentially expressed at the transcript level in the V0&V15, V15&V30, and V0&V30 comparisons, respectively. Correspondingly, 42, 9, and 32 TFs were differentially expressed at the protein level (Table S3). The predominant TF families responding to vernalization included Myeloblastosis (MYB), Ethylene Response Factor (ERF), basic Helix-Loop-Helix (bHLH), WRKY, basic Leucine Zipper (bZIP), and NAM, ATAF1/2, and CUC2 (NAC) (Fig. S4A). Notably, 9 and 13 TFs overlapped between the DEGs and DEPs in the V0&V15 and V0&V30 comparisons, respectively (Fig. 3E). While the transcript levels of most overlapping TFs decreased with prolonged vernalization, the protein abundance of some displayed an inverse trend (Fig. 3F and Fig. S4B-S4C). This discrepancy suggests that active post-transcriptional or post-translational regulation modulates these key regulatory factors during vernalization. Finally, the expression trends of selected TFs were validated by qRT-PCR, confirming their consistency with the RNA-seq data (Fig. 3G).

### Integrated transcriptome and proteome analysis

**Fig. 4:**
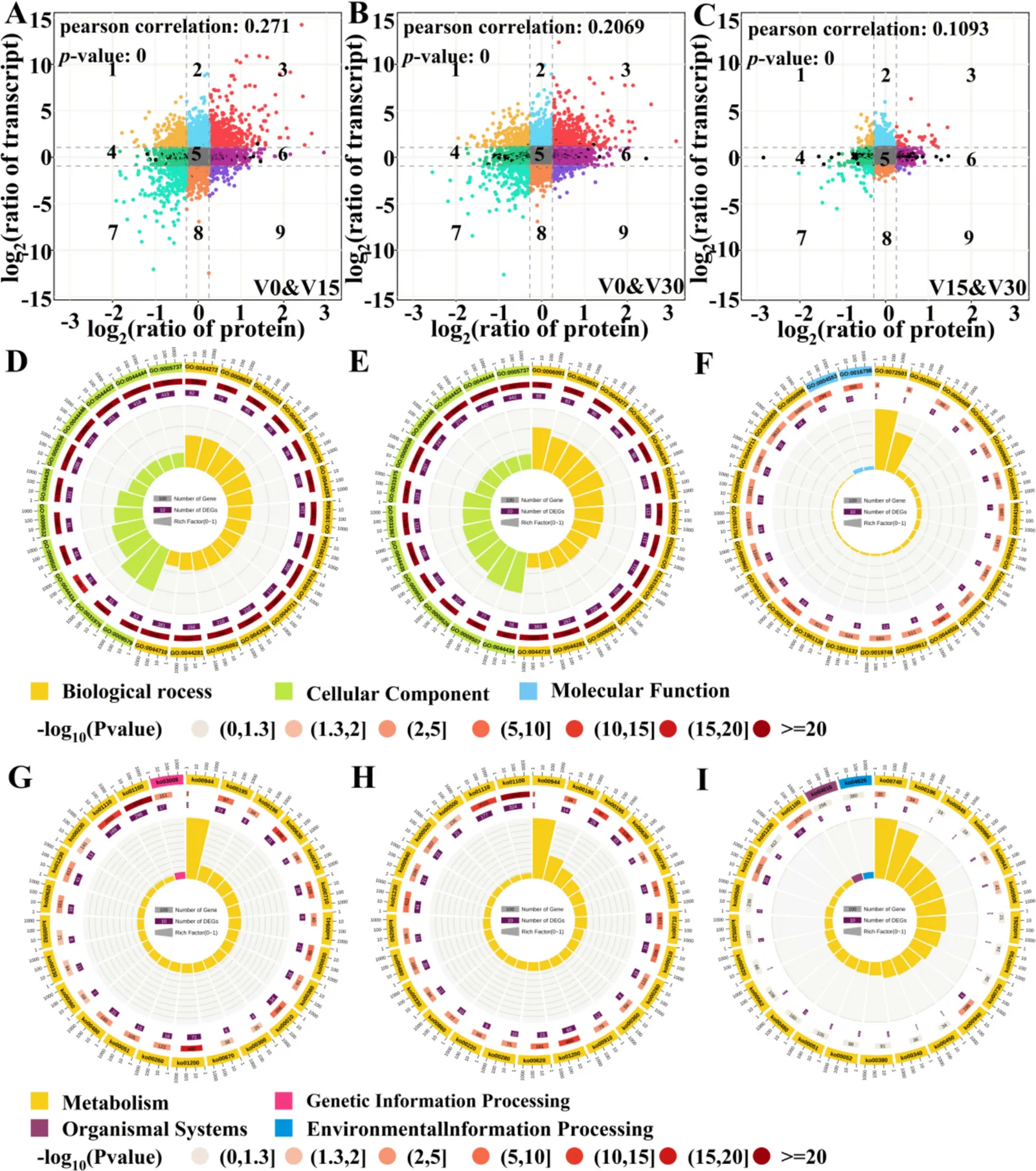
Nine-quadrant, GO, and KEGG analyses. **A-C** Nine-quadrant analysis of mRNA-protein pairs for V0&V15 (**A**), V0&V30 (**B**), and V15&V30 (**C**); each dot represents a matched mRNA-protein pair; quadrants are numbered 1–9; quadrants 1, 2, and 4 indicate higher transcript abundance relative to protein levels; quadrants 3 and 7 represent a positive correlation between mRNA and protein levels; quadrants 6, 8, and 9 indicate higher protein abundance relative to mRNA levels; quadrant 5 contains co-expressed but non-differentially expressed mRNA–protein pairs; black dot represent pairs with non-significant changes at both levels. **D-F** GO enrichment analysis of genes/proteins annotated in the quadrants 1, 3, 7, and 9 (*p* < 0.05) in the V0&V15 (**D**), V0&V30 (**E**), and V15&V30 (**F**) comparisons. **G-I** KEGG pathway enrichment analysis of genes/proteins annotated in the quadrants 1, 3, 7, and 9 (*p* < 0.05) in the V0&V15 (**G**), V0&V30 (**H**), and V15&V30 (**I**) comparisons

In total, 7,774, 7,730, and 7,741 mRNA–protein pairs were identified in the V0&V15, V0&V30, and V15&V30 comparisons, respectively (Fig. S3A-S3C). To assess the coordination between transcript and protein levels, a nine-quadrant analysis was performed, which revealed distinct correlation patterns across the vernalization stages (Fig. 4A-C). Notably, approximately 40% of mRNA–protein pairs were annotated into quadrant 5 (indicating non-differentially expressed pairs) in the V0&V15 and V0&V30 comparisons, whereas this proportion exceeded 80% in the V15&V30 comparison (Fig. S5). Furthermore, the number of transcript/protein pairs exhibiting concordant differential abundance—defined as a ≥ 2-fold change in transcript levels concomitant with a ≥ 1.2-fold change in protein abundance—was similar between the V0&V15 and V0&V30 comparisons, but was significantly lower in the V15&V30 group (Fig. 4A-C and S5). These results indicate that most vernalization-responsive genes undergo transcriptional and proteomic adjustments primarily during the initial low-temperature exposure. These altered expression states are then maintained during subsequent vernalization stages.

**Fig. 5:**
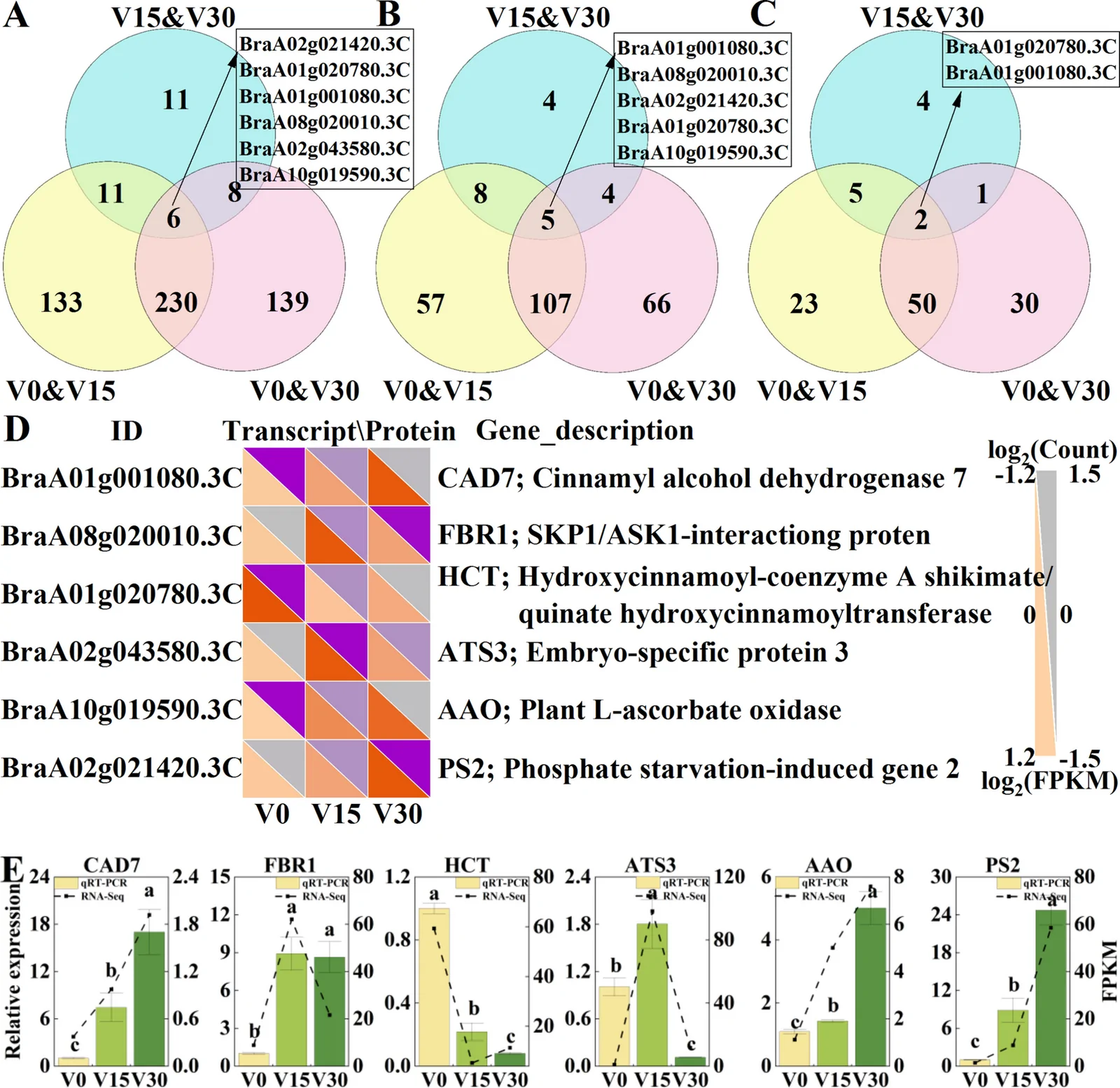
Gene/protein pairs in the single-organism biosynthetic and metabolic, and secondary metabolic processes. **A-C** Venn diagrams showing the distribution of gene/protein pairs associated with the single-organism biosynthetic process (**A**), single-organism metabolic process (**B**), and secondary metabolic process (**C**) across the three comparison groups. **D** Heatmap of transcript and protein expression levels; row scales were normalized and the values were scaled by log2; heatmap colors indicate FPKM (mRNA) or count (protein), and the darker the color, the higher the level. **E** Relative expression analysis based on qRT-PCR (bar chart, left Y-axis) and RNA-Seq (line chart, right Y-axis); error bars indicate the means ± SEs; different letters above the bars indicate significant differences between different stages, determined by Duncan’s test at *p* < 0.05

GO and KEGG enrichment analyses (*p* < 0.05) were performed for genes/proteins in quadrants 1, 3, 7, and 9 (Fig. 4D-I; Tables S4 and S5). Biological process (BP) and cellular component (CC) dominated the enriched GO terms. Key BP terms included single-organism biosynthetic process (GO:0044711), single-organism metabolic process (GO:0044710), and secondary metabolic process (GO:0019748), which were common to all comparison groups (Fig. 5A-C and S6; Table S4). Within these processes, six genes/proteins (*CAD7*, *FBR1*, *HCT*, *ATS3*, *AAO*, and *PS2*) were identified (Fig. 5D-E).

KEGG enrichment analysis revealed significant enrichment in metabolic-related processes. The metabolic pathways (ko01100), biosynthesis of secondary metabolites (ko01110), and flavonoid biosynthesis (ko00941) were consistently enriched across all groups (Fig. 4G-I and Fig. S7; Table S5). Pathways related to amino acid biosynthesis, metabolism, and degradation were specifically enriched in the V0&V15 and V0&V30 comparisons (Fig. S7A). Amino sugar and nucleotide sugar metabolism (ko00520), arginine biosynthesis (ko00220), and alanine, aspartate and glutamate metabolism (ko00250) were specifically enriched in V0&V30, while lysine biosynthesis (ko00300) was unique to V0&V15 (Fig. S7A; Table S5). Collectively, these results provide a multidimensional profile of the metabolic and regulatory pathways associated with vernalization at both the transcript and protein level.

### Regulation of sugar metabolism during vernalization

**Fig. 6:**
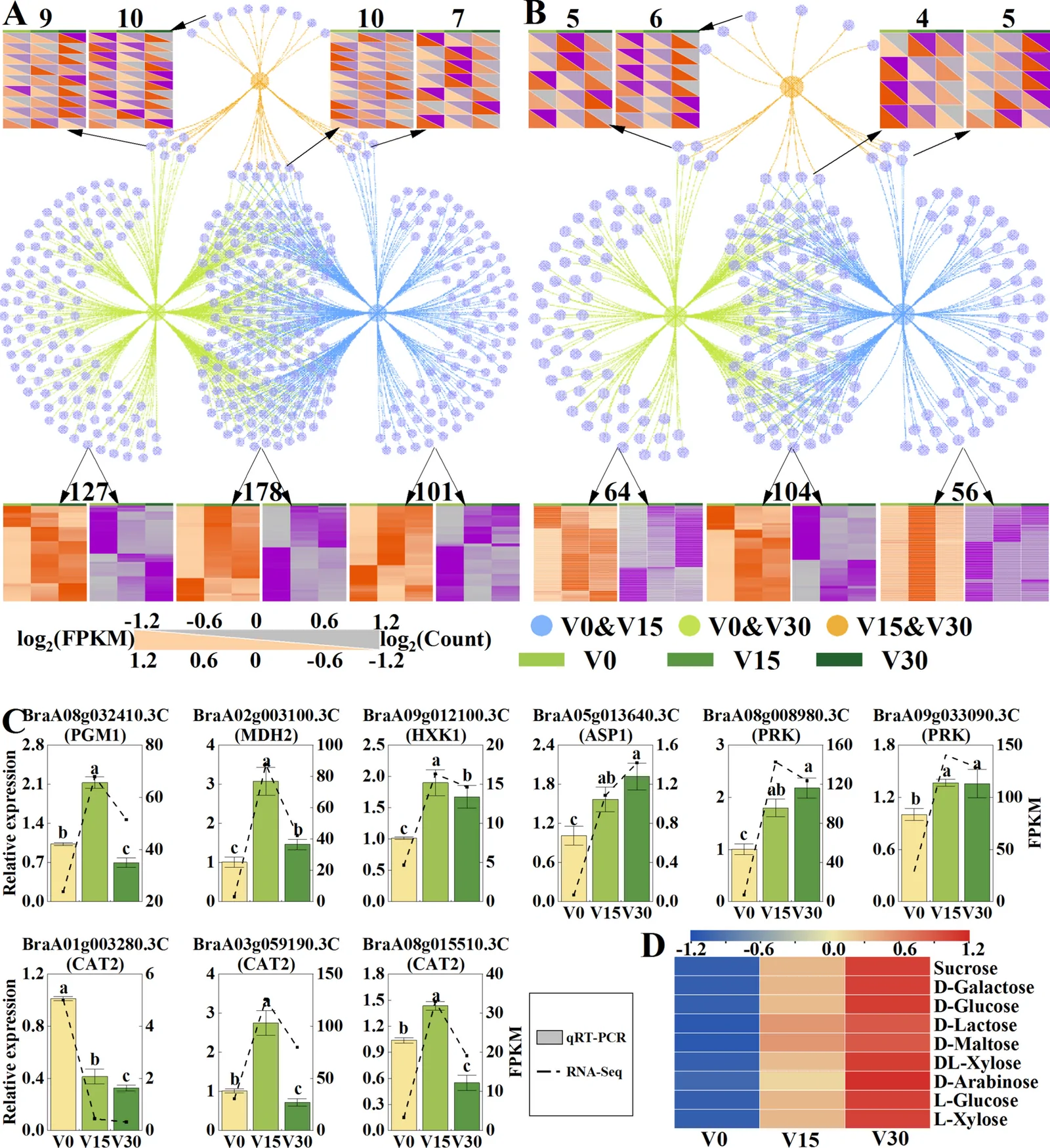
Gene/protein pairs in the KEGG pathway of metabolism, and of secondary metabolite biosynthesis. **A**, **B** Venn and heatmap diagrams of gene/protein pairs enriched in the metabolic pathway (ko01100; **A**) and biosynthesis of secondary metabolites pathway (ko01110; **B**); row scales were normalized and the values were scaled by log2; heatmap colors indicate FPKM (mRNA) or count (protein), and the darker the color, the higher the level; total number of genes/proteins displayed is indicated above each heatmap; the dots and rectangles below the heatmaps represent different comparison groups and different vernalization periods, respectively. **C** Relative expression analysis based on qRT-PCR (bar chart, left Y-axis) and RNA-Seq (line chart, right Y-axis); error bars indicate the means ± SEs; different letters above the bars indicate significant differences between different stages, as determined by Duncan’s test at *p* < 0.05. **D** Heatmap of sugar contents; heatmap colors indicate normalized sugar content (log_2_); red and blue indicate high and low abundance, respectively

Within quadrants 1, 3, 7, and 9, 442 and 244 genes/proteins were annotated to metabolic pathways (ko01100) and biosynthesis of secondary metabolites (ko01110), respectively (Fig. S7B-S7C; Table S6). Notably, most of these genes exhibited upregulated transcripts but downregulated protein abundance (Fig. 6A-B). Carbon metabolism is known to play a critical role in vernalization (Fusco et al. 2023; Sun et al. 2015), and in this study, all genes in the carbon metabolism pathway were also enriched in the pathways of metabolism and secondary metabolites biosynthesis (Fig. S7D). Furthermore, numerous genes involved in sucrose response and sucrose-mediated signaling pathways were up-regulated during vernalization (Fig. 6C and Fig. S7E). Consistent with these transcriptional changes, we observed a significant accumulation of sugars, including sucrose, glucose, galactose, lactose, maltose, xylose, and arabinose, as well as total soluble sugars, throughout the vernalization process (Fig. 6D). The activities of sucrose synthase and sucrose phosphate synthase gradually increased during the vernalization (Fig. S8). These coordinated transcriptomic and metabolic findings strongly suggest a key role for enhanced carbon metabolism and mobilization in the vernalization response of Wucai.

### Regulation of amino acid metabolism during vernalization

**Fig. 7:**
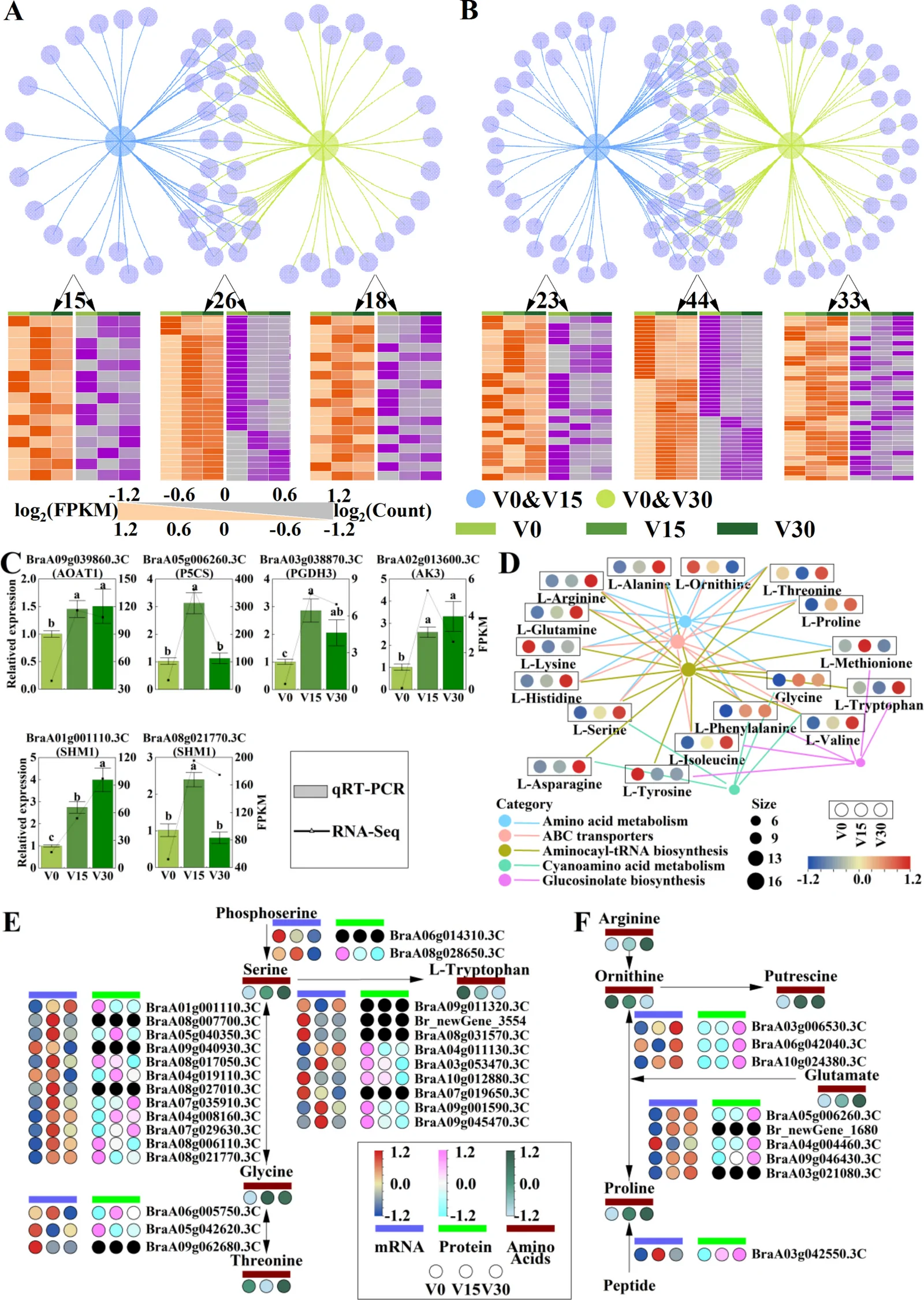
Amino acid biosynthesis and metabolism. **A**, **B** Venn and heatmap diagrams of gene/protein pairs enriched in the amino acid biosynthesis (ko01230; **A**) and various amino acid metabolism pathways (ko00380, ko00260, ko00480, ko00330, ko00360, ko00300, ko00280, ko00250, ko00220; **B**); row scales were normalized and the values were scaled by log2; heatmap colors indicate FPKM (mRNA) or count (protein), and the darker the color, the higher the level; the numbers above the heatmap indicate the gene/protein number; the dots and rectangles below the heatmap represent different comparison groups and vernalization periods, respectively. **C** Relative expression analysis based on qRT-PCR (bar chart, left Y-axis) and RNA-Seq (line chart, right Y-axis); error bars indicate the means ± SEs; different letters above the bars indicate significant differences between different stages, as determined by Duncan’s test at *p* < 0.05. **D** KEGG network of amino acid metabolites; node colors represent different pathways, and the connected boxes are specific metabolites annotated to the pathway; heatmaps in the boxes indicate normalized content (log_2_); red and blue indicate high and low abundance, respectively; internal circular dots represent different vernalization periods. **E**, **F** Pathway maps of glycine, serine and threonine metabolism (**E**), and arginine and proline metabolism (**F**); heatmap colors indicate normalized mRNA transcript, protein abundance, and amino acid content (log2), respectively; the darker the color, the higher the level; black dots indicate undetected values; internal circular dots represent different vernalization periods

Amino acid metabolism pathways were significantly enriched (Table S5), including biosynthesis (ko01230) and multiple metabolism pathways (ko00380, ko00260, ko00480, ko00330, ko00360, ko00300, ko00280, ko00250, ko00220). A total of 26 genes were involved in amino acid biosynthesis, and 44 in multiple amino acid metabolism pathways between V0&V15 and V0&V30 (Fig. 7A-B). The majority of these genes showed upregulated transcript levels coupled with reduced protein abundance during vernalization. Among these, eight key genes involved in glycine, serine and threonine metabolism (ko00260) and arginine and proline metabolism (ko00330) were identified (Fig. 7C and Fig. S9, Table S7). Notably, BraA08g032410.3C (*PGM1*) and BraA05g013640.3C (*ASP1*) were also involved in the carbon metabolism (ko01200) pathway (Fig. 6C and Table S8), suggesting a potential crosstalk between carbon and nitrogen metabolism.

Metabolomic profiling identified 26 differentially accumulated amino acid metabolites across all comparison groups (Fig. S10A-S10C). The majority of these metabolites accumulated progressively during vernalization (Fig. S10D), and were involved in key pathways including amino acid metabolism, ABC transporters, aminoacyl-tRNA biosynthesis, cyanoamino acid metabolism, and glucosinolate biosynthesis pathways (Fig. 7D). Notably, glycine, serine, proline, and arginine showed substantial enrichment during vernalization (Fig. S10E-S10I), and their corresponding genes were consistently upregulated at both transcript and protein levels (Fig. 7E-F), underscoring their potential critical roles in mediating the vernalization-induced flowering transition. Furthermore, exogenous proline treatment significantly upregulated the expression of *BcFT* and *BcSOC1*, consequently accelerating bolting in Wucai (Fig. S11).

### Regulation of hormone metabolism during vernalization

**Fig. 8:**
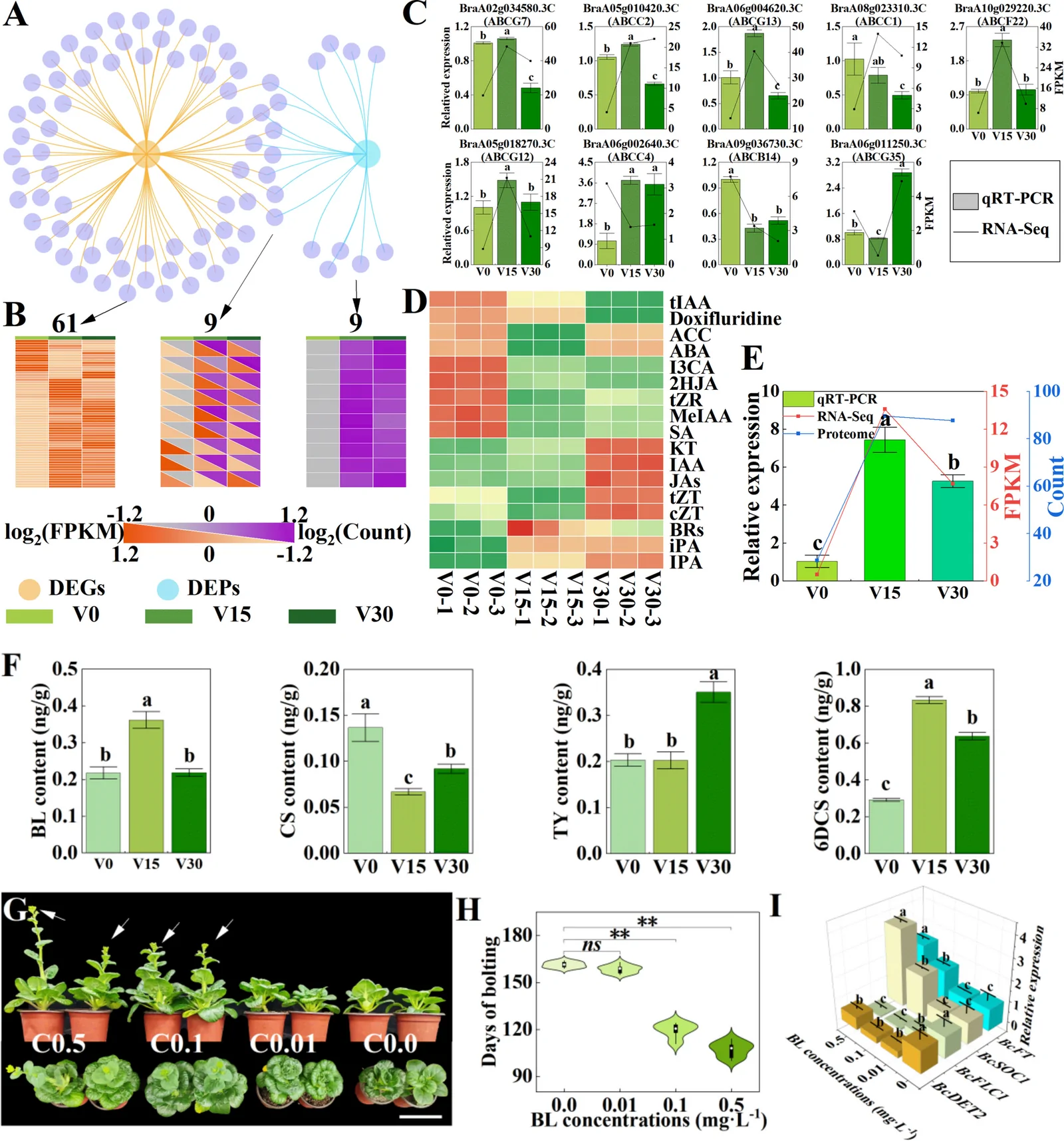
ABC transporters and phytohormone dynamics. **A**, **B** Venn (**A**) and heatmap (**B**) diagrams of ABC transporter DEGs/DEPs; row scales were normalized and the values were scaled by log2; heatmap colors indicate FPKM (mRNA) or count (protein), and the darker the color, the higher the level; the numbers above the heatmap indicate the gene/protein number; the dots and rectangles below the heatmap represent different comparison groups and vernalization periods, respectively. **C** Relative expression analysis of ABC transporter genes based on qRT-PCR (bar chart, left Y-axis) and RNA-Seq (line chart, right Y-axis). **D** Phytohormone content heatmap; 2HJA, dihydrojasmonic acid; ABA, abscisic acid; ACC, 1-aminocyclopropanecarboxylic acid; BRs, brassinolides; cZT, cis-zeatin; tZT, trans-zeatin; tIAA, trans-indole-3-acrylic acid; tZR, trans-zeatin-riboside; I3CA, indole-3-carboxaldehyde; IAA, indole-3-acetic acid; iPA, N6-isopentenyladenine; IPA, isopentenyl adenosine; MeIAA, methyl indole-3-acetate; JA, (±)-jasmonic acid; KT, kinetin; SA, salicylic acid; row scales were normalized and the values were scaled by log2; heatmap colors indicate phytohormones contents, and the darker the color, the higher the level. **E** Relative expression of *BcDET2* gene based on qRT-PCR (bar chart, left Y-axis) and RNA-Seq (red line chart, right Y-axis), and protein levels (blue line chart, right Y-axis) during vernalization. **F** Endogenous concentrations of BRs during vernalization; BL, brassinolide; CS, castasterone; 6DCS, 6-deoxocastasterone; TY, typhasterol. **G** Representative plant phenotypes following treatment with different concentrations of BL; C0.0, C0.01, C0.1, and C0.5 represent BR concentrations of 0.0, 0.01, 0.1, and 0.5 mg/L, respectively; white arrows indicate early flowering after spraying BR at concentrations of 0.1, and 0.5 mg/L; images were taken approximately120 days after planting; scale *bar* = 10 cm. **H** Bolting time under different BL treatment, *n* = 10. **I** Expression of *BcDET2*, *BcFLC1*, *BcSOC1*, and *BcFT1* under BL treatments. Error bars indicate the means ± SEs; different letters above the bars indicate significant differences between different stages, as determined by Duncan’s test at *p* < 0.05; the asterisks indicate the significant differences at *p* < 0.01 (**) by Student’s test; *ns*, no significant

Our analysis identified 129 ABC transporter members expressed at transcriptomic or proteomic levels (Table S9). Among these, 56 were detected only at the transcriptomic level with low expression, while 27 were detected at both transcript and protein levels (Fig. S12A). Differential expression analysis revealed 70 ABC transporters significantly regulated at the transcriptomic level and 18 at the proteomic level, with 9 overlapping between the two datasets (Fig. 8A). Most of these were upregulated at the transcript level (Fig. 8B-C). Given the established role of ABC transporters in phytohormone transport (Do et al. 2018), we quantified endogenous hormone levels and identified 17 phytohormones that were significantly altered during vernalization (Fig. S12B-S12D; Table S10). The levels of tIAA, ACC, and SA were decreased, whereas KT, IAA, JAs, ZTs, and BRs increased significantly (Fig. 8D). These results suggested that multiple hormonal pathways are involved in regulating the vernalization response in Wucai.

Given that previous reports implicated BRs in floral transition (Li et al. 2024, 2018), we further focused on their biosynthesis pathway. Among the BR biosynthesis genes, only *BcDET2*, a key rate-limiting enzyme, showed significantly increased expression at both transcript and protein levels (Fig. 8E and Fig. S13; Table S11). Consistently, the endogenous levels of several BRs, including BL, TY, and 6DCS increased during vernalization (Fig. 8F). To functionally validate this finding, we applied exogenous BR and found that it significantly upregulated the expression of *BcFT* and *BcSOC1*, consequently accelerating bolting and flowering in Wucai (Fig. 8G-I). Functional validation of *BcDET2* in *Arabidopsis* revealed that its overexpression increased endogenous BR levels and thereby accelerated flowering (Fig. S14). Together, these results demonstrate that *BcDET2* is a key regulator of BR-mediated vernalization and floral induction.

## Discussion

### Vernalization pathway genes respond to vernalization

Similar to other *Brassica* crops, Wucai requires prolonged exposure to low temperatures to initiate bolting and flowering, a process that promotes the transition from vegetative to reproductive development (Dai et al. 2024; Liu et al. 2025). In *Arabidopsis*, more than 180 genes have been identified as involved in flowering regulation (Wu et al. 2024), including key vernalization pathway regulators such as *FRI*, *VRN*, *VIN*, *FLC*, and *SVP* (Wu et al. 2024). The MADS-box transcription factor *FLC* acts as a central repressor, delaying flowering by directly suppressing the expression of floral integrators like *SOC1* and *FT*, thereby inhibiting *FD* upregulation in the SAM (Li et al. 2022). During vernalization, cold-induced expression of *VIN3* and *VIL1* facilitates the polycomb-mediated epigenetic silencing of *FLC*/*FLM* via histone methylation/deacetylation, while VRN proteins maintain the vernalization state (Gao et al. 2022; Hepworth et al. 2018; Li et al. 2020). *VIN3-LIKE 2* (*VIL2*) is also involved in this process by silencing the *MAF5* gene (Salomé, 2022).

In this study, we identified 28 vernalization-pathway genes in the shoot apices of Wucai, a tissue critical for perceiving the cold signal (Dai et al. 2024; Lazaro et al. 2019). Among these, there was single copy of *SVP* and *VIL2*, two copies each of *FD*, *FLM/MAF*, and *SOC1*, and three copies of *FLC*, *VIN3*, and *VRN* (Fig. 3A-B). This gene multiplicity is attributed to whole-genome triplication (WGT) during evolution (Cai et al. 2017). During vernalization, their expression dynamics were largely consistent with the established model: *FLC*, *FLM*, and *SVP* homologs were downregulated, while *VIN3* and *VIL2* were upregulated (Fig. 3B and D), consistent with previous reports in other species (Kang et al. 2022; Kim et al. 2022). However, these duplicated copies had divergent expression patterns and responses to vernalization (Fig. 3A-B), suggesting subfunctionalization may fine-tune the cold-response threshold. Although *VRN1* has been proposed as a major initiator of the regulatory cascade leading to flowering and promoting the transition of the SAM to the reproductive phase (Liu et al. 2024a), both transcript and protein levels of *VRN1* were downregulated during vernalization in our study (Fig. 3C-D). This could indicate functional divergence of Wucai VRN1 paralogs, with canonical *VRN1s* activity potentially compensated by other factors or these paralogs recruited for a novel role in cold adaptation, which warrants further investigation.

### Transcription factors modulate vernalization responses

Transcription factors play pivotal roles in regulating flowering during vernalization. Various TF families such as MADS, MYB, bHLH, WRKY, GRAS, bZIP, C2H2, and NAC have been shown to regulate flowering by either activating or repressing gene expression during vernalization (Wang et al. 2023a; Zeng et al. 2025). Similarly, we identified MYB, bHLH, WRKY, ERF, and bZIP as the predominant vernalization-responsive TF families in Wucai (Fig. S4A; Table S3), consistent with findings in other vernalization-requiring crops (Dai et al. 2024; Liu et al. 2024b; Shi et al. 2021). These TFs forms a complex regulatory network that modulates the vernalization pathway. For instance, in *Arabidopsis*, *AtMYB106* acts as a floral repressor by inhibiting *AtFT* expression (Hong et al. 2021b), and *MYBH*, another repressor, is involved in the GA response (Slotte et al. 2007). In our analysis, a MYBH homolog (BrA01g019220.3C) was differentially expressed during vernalization (Fig. 3E-F). Furthermore, MYB57 and bHLH3, which participate in the JA signaling pathway (Zhao et al. 2022, 2023), were downregulated at both transcript and protein levels (Fig. 3E-F). Additionally, bZIP and NF-YC family members, known to influence floral organ development and flowering (Martignago et al. 2023; Zhang et al. 2023a), were upregulated. Specifically, we observed upregulation of ABF4 (BraA05g027980.3C), bZIP59 (BraA04g022790.3C), and NF-YC9 (BraA06g005970.3C) during vernalization (Fig. 3E-F). Given that *Arabidopsis ABF4* interacts with *NF-YC9* to regulate *SOC1* expression and mediate stress-accelerated flowering (Hwang et al. 2019), the upregulation of their homologs in Wucai suggests a conserved and critical regulatory role in the vernalization response.

### Alterations in carbohydrate metabolism during vernalization

Low temperatures modulate gene expression to regulate various metabolic and signaling pathways, ultimately influencing the floral transition (Sun et al. 2015). In this study, genes and proteins with consistent or decoupled expression patterns (DEGs and DEPs in the 1, 3, 7, and 9 quadrants) were significantly enriched in GO terms such as single-organism biosynthetic and metabolic processes, and secondary metabolic process (Fig. 5A; Table S4). These elements were also enriched in key KEGG pathways, including metabolism and secondary metabolite biosynthesis (Fig. 6A; Table S5). Similar observations have been reported in other vernalized crops (Gu et al. 2020; Nugroho et al. 2021; Wang et al. 2023a). Within this metabolic reprogramming, carbohydrate metabolism emerged as a primary pathway activated during vernalization. Notably, sucrose signaling and sucrose-mediated response pathways were prominently featured (Fig. S7D). We found that numerous genes involved in glucose metabolism and sucrose-mediated signaling were upregulated under vernalization (Fig. 6C and Fig. S7E). This transcriptional upregulation suggests a promotion of sucrose-related pathways during vernalization. Low temperature vernalization triggers a systemic modification of photosynthesis, photorespiration, and respiratory metabolism, which is thought to stimulate the supply and transport of photosynthates such as sucrose to the shoot apex (Hüner et al. 2016; Liu et al. 2025). This premise is strongly supported by our metabolomic data, which revealed significant accumulation of sucrose, soluble sugars, glucose, galactose, lactose, maltose, xylose, and arabinose (Fig. 6D). Similar sugar accumulation has been documented in various vernalized species (Fusco et al. 2023; Rodrigues et al. 2020), collectively indicating that enhanced carbohydrate metabolism and mobilization play a key role in regulating plant developmental transitions during vernalization (Fusco et al. 2023; Sun et al. 2015).

### Alterations in amino acid metabolism during vernalization

Vernalization accelerates the transition from vegetative to reproductive growth and significantly alters plant metabolite profiles (Liu et al. 2025; Nugroho et al. 2021). In our study, multi-omics analysis revealed that multiple amino acid metabolic pathways were significantly changed during vernalization (Fig. 4G-I and Fig. S7A; Table S5). This was accompanied by a pronounced accumulation of specific amino acids, including glycine, serine, proline, and arginine (Fig. S10E-S10I). Such accumulation may serve as a potential signaling event that promotes the floral transition (Carillo et al. 2019), a phenomenon also observed in other vernalization-responsive species such as *Ranunculus asiaticus*, *Isatis indigotica*, and *Brassica napus* (Fusco et al. 2023; James et al. 2025; Wang et al. 2021). Among these amino acids, proline is of particular interest due to its demonstrated role in influencing *FLC* expression via epigenetic regulation (Mattioli et al. 2022). For instance, increased proline content in *P5CS1*-overexpressing *Arabidopsis* leads to early flowering (Mattioli et al. 2009). In Wucai, we observed a simultaneous upregulation of *P5CS* expression and proline accumulation during vernalization (Fig. 7C-D). Beyond simply altering amino acid content, vernalization appears to reform the underlying metabolic pathways (Fusco et al. 2023). Our data confirm this, showing significant changes at both the transcript and protein levels of key genes in the glycine, serine, and proline metabolic pathways (Fig. 7E-F). Specifically, the expression of several amino acid synthase-related genes was significantly changed during vernalization (Fig. 7C, Table S8). These findings indicate that vernalization modulates amino acid accumulation, at least in part, through the transcriptional and translational regulation of biosynthetic enzymes (Hosseini et al. 2023).

### Alterations in plant hormone metabolism during vernalization

Vernalization induces comprehensive metabolic reprogramming, affecting sugar and free amino acids as well as the homeostasis of endogenous hormones and the expression of hormone-related genes (Liu et al. 2022). These hormonal signaling pathways are crucial components of the regulatory network controlling the floral transition (Campos-Rivero et al. 2017). In Wucai, our integrated analysis revealed that multiple free amino acids participate in ABC transporter pathways (Fig. 7D), with the majority of ABC transporter genes being upregulated (Fig. 8A-C; Table S9). Furthermore, key genes related to the biosynthesis and signaling of gibberellic acid, ethylene, abscisic acid, and jasmonic acid pathways were enriched during vernalization (Wang et al. 2023a, b; Shi et al. 2021). A phenomenon also reported in *Angelica sinensis*, where dynamic changes in metabolites and hormones occur during vernalization (Liu et al. 2022). Our hormone profiling confirmed significant alterations in most phytohormones (Table S10), indicating their roles during vernalization in Wucai. Interestingly, while previous studies have reported changes in GA levels during vernalization (Shang et al. 2017; Tilmes et al. 2019), our data did not show significant GA fluctuations. Instead, we observed an increase in IAA, KT, JA, and BRs (Fig. 8D). Given the established role of BR signaling in modulating flowering time (Cui et al. 2023), we focused on this pathway. This study found that *DET2*, a key enzyme in BR biosynthesis, was upregulated at both the transcript and protein levels (Fig. 8E). This upregulation corresponded with a significant increase in the endogenous levels of specific BRs, including BL, TY, and 6DCS (Fig. 8F). The biological relevance of BR accumulation is supported by evidence from *Arabidopsis thaliana*, where mutants with impaired BR biosynthesis or signaling (*det2*, *dwf4*, and *bri1*) delayed flowering (Cui et al. 2023). To test BR function in Wucai, we performed exogenous BR application assays. BR treatment suppressed the expression of the floral repressor *FLC,* while enhancing the expression of the floral integrators *FT* and *SOC1*, ultimately accelerating flowering (Fig. 8G-I). Consistent with this, heterologous expression of *BcDET2* in *Arabidopsis thaliana* yielded analogous results (Fig. S14). Collectively, these findings demonstrate that vernalization-induced hormonal changes facilitate the transition from vegetative to reproductive growth in Wucai.

## Conclusions

**Fig. 9:**
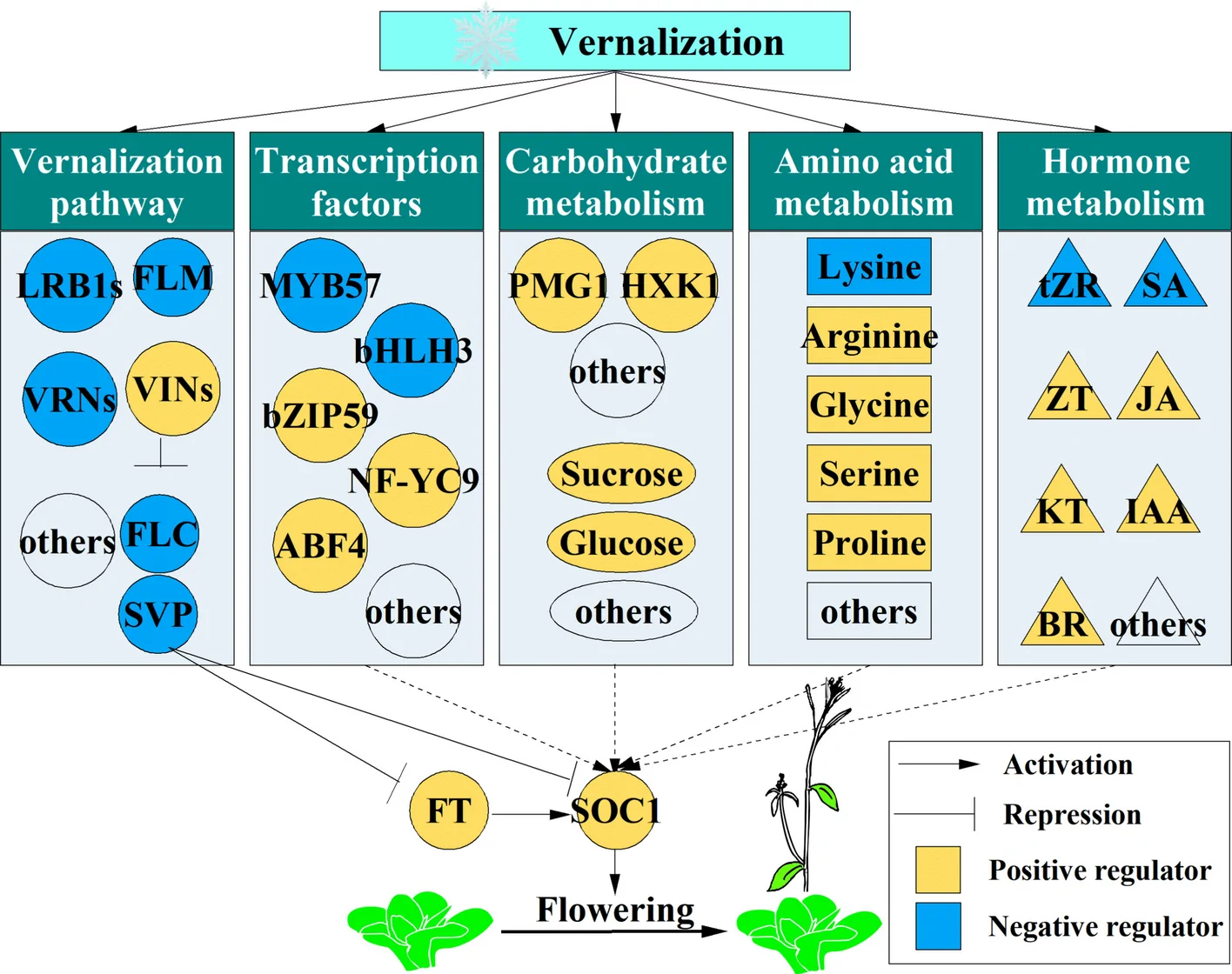
Vernalization mediates flowering in Wucai. Prolonged cold exposure promotes flowering through the core vernalization pathway and interconnected metabolic pathways, including sugar metabolism, amino acid metabolism, and hormone signaling

This study elucidates the vernalization-responsive regulatory network in Wucai by integrating transcriptomic, proteomic, and metabolomic analysis. We identified the conserved repression of *FLC* homologs and induction of *VIN3*, alongside divergent *VRN1* regulation, suggesting potential differences in vernalization mechanisms in Wucai. Multiple transcription factor families, including MYB, bHLH, bZIP, and NF-YC, exhibited differential expression, highlighting their roles in coordinating vernalization-responsive gene networks. In addition to transcriptional regulation, vernalization triggered extensive metabolic adjustments, characterized by significant accumulation of sucrose and amino acids (proline, glycine, serine) driven by coordinated upregulation of their biosynthetic pathways. Hormonal profiling revealed elevated endogenous levels of IAA, JAs, KT, and BRs. Notably, vernalization enhanced BR biosynthesis via upregulation of *DET2*, and exogenous BR application accelerated flowering by repressing *FLC* and activating floral integrators *FT* and *SOC1*. Overall, our findings provide a comprehensive insight into the complex transcriptional, metabolic, and hormonal networks underlying vernalization in Wucai (Fig. 9), offering promising molecular targets for modulating flowering time and enhancing climate resilience in *Brassica* crops.

## Materials and methods

### Plant materials and vernalization treatment

Thirty-day-old seedlings of the Wucai inbred line “HW” were utilized as plant materials in this study. Seedlings were vernalized in a growth chamber at 4 °C under a 16 h light/8 h dark photoperiod. Samples of shoot apices (SA; including the shoot apical meristem and primordial leaves) were harvested after 0, 15, and 30 days of vernalization treatment (designated V0, V15, and V30, respectively; Fig. 1A-C). The collected tissues were immediately frozen in liquid nitrogen and stored at −80 °C until analysis.

### Transcriptomic profiling

Total RNA was extracted from the samples using TRIzol reagent (Invitrogen, USA) following the manufacturer’s protocol. cDNA libraries were prepared with the NEBNext Ultra™ Directional RNA Library Prep Kit for Illumina (NEB, USA) and sequenced on an Illumina Hi-Seq platform by Biomarker Technologies Co., Ltd (Beijing, China). The experiment included three biological replicates per time point. Differentially expressed genes (DEGs) were identified using DESeq2 with thresholds of |fold change (FC)|≥ 2 and false discovery rate (FDR) < 0.05.

### TMT labeling and proteome analysis

Proteins were extracted using the trichloroacetic acid (TCA)/acetone method (Liu et al. 2025). Protein concentration was measured using the Bradford Protein Assay Kit (Bio-Rad, Hercules, CA, USA), and quality was verified by sodium dodecyl sulfate polyacrylamide gel electrophoresis (SDS-PAGE). For each sample, 100 μg of protein was digested with Trypsin Gold (Promega, USA) and labeled with tandem mass tags (TMT) reagent (Thermo Fisher Scientific, USA). The labeled peptides were incubated with the Pierce High pH Reversed-phase Fractionation Kit (Thermo Fisher Scientific, USA), and analyzed by High-Performance Liquid Chromatography-tandem Mass Spectrometer (HPLC–MS/MS). Data were processed using Protein Pilot Software V5.0 (Sciex, USA), retaining proteins identified at a global FDR ≤ 1% and with unique peptides ≥ 1. Differentially expressed proteins (DEPs) were identified based on the criteria of FC > 1.2 and *p* < 0.05.

### Integrated multi-omics analysis

Functional annotations for genes, proteins, and metabolites were assigned using Gene Ontology (GO), Kyoto Encyclopedia of Genes and Genomes (KEGG), KEGG Orthology (KO), Swiss-Prot, and non-redundant protein (Nr) databases. Enrichment significance for GO terms and KEGG pathways among DEGs/DEPs was determined (*p* < 0.05). Correlation analysis between DEGs and DEPs was conducted using a nine-quadrant analysis via the OmicShare tools platform (www.omicshare.com).

### Real-time quantitative PCR (qRT-PCR) validation

qRT-PCR was performed using SYBR Premix Ex Taq (TaKaRa, Japan) on a Bio-Rad CFX96™ Real-Time PCR Detection System using gene-specific primers (Table S1). *BcActin1* served as an internal reference gene (Chen et al. 2018; 2020). Each qRT-PCR assay included three biological replicates and three technical replicates. Relative gene expression levels were calculated using the 2^−ΔΔCt^ method.

### Quantification of sugar and free amino acids

The extraction and quantification of sugars and free amino acids were performed as previously described (Liang et al. 2022). Approximately 10 mg shoot apical samples were used for metabolite extraction. Free amino acids were extracted with a methanol: acetonitrile: water (2:2:1, v/v/v) solution containing isotopically labeled internal standards. Sugar and other metabolites were extracted with 70% methanol, while phytohormones were extracted with 80% isopropyl alcohol containing internal standards. The supernatants were filtered through a 0.22 μm membrane and analyzed by Ultra-High Performance Liquid Chromatography-Multiple Reaction Monitoring-Tandem Mass Spectrometry (UHPLC-MRM-MS/MS) platform (UHPLC, Waters ACQUITY I-Class, Waters Technology; MS, SCIEX QTRAP 6500 +, SCIEX).

### Brassinosteroid quantification

Endogenous brassinosteroids (BRs), including brassinolide (BL), castasterone (CS), 6-deoxocastasterone (6DCS), and typhasterol (TY), were quantified using an HPLC–MS/MS system according to the previous methods (Wang et al. 2023b). The analysis was performed with three biological replicates, each comprising three technical replicates.

### Exogenous brassinosteroid treatment

Thirty-day-old Wucai seedlings were utilized for exogenous BR treatment. Aqueous solutions of 24-Epibrassinolide (Sigma) at concentrations of 0, 0.01, 0.1, and 0.5 mg/L were sprayed onto fully expanded leaves at night. The treatment was repeated once every 7 days for a total of five applications (Chen et al. 2025). At flowering onset, transcript levels of flowering-related genes were quantified to assess BR-induced gene expression changes.

### Statistical analysis and data visualization

Statistical analysis was performed using one-way ANOVA followed by Duncan’s multiple range test (*p* < 0.05) in SPSS 20.0 (IBM, USA). Data visualizations, including Venn diagrams and heatmaps, were generated using TBtools-II, BMK Cloud platform (www.biocloud.net/), and OmicShare tools (www.omicshare.com).

## Supplementary Information

Supplementary Material 1: Figures. Fig. S1. Quality assessment of RNA-Seq data and statistics of proteomic identifications. Fig. S2. Pearson’s correlation coefficients and principal component analyses. Fig. S3. Concordant tests between proteins and cognate mRNAs. Fig. S4. Statistics and expression trends of the TFs. Fig. S5. Distribution of transcript/protein pairs across a nine-quadrant plots. Fig. S6. GO enrichment of biological processes for gene/protein pairs in the quadrants 1, 3, 7, and 9. Fig. S7. Venn diagrams of enriched KEGG pathways. Fig. S8. Activities of sucrose synthase (A) and sucrose phosphate synthase (B) during vernalization process. Fig. S9. Genes involved in amino acid biosynthesis and metabolism. Fig. S10. Assay of amino acid metabolites. Fig. S11. Representative plant phenotypes following treatment with different concentrations of proline. Fig. S12. Analysis of ABC transporters and phytohormone abundance. Fig. S13. Expression of BR biosynthesis genes. Fig. S14. Functional characterization of BcDET2 in Arabidopsis.

Supplementary Material 2: Tables. Table S1 Primer sequences used for qRT-PCR analysis. Table S2. Shared DEGs and DEPs were identified and trend expression analysis. Table S3. Identified transcription factors among DEGs and DEPs. Table S4. Significantly enriched GO biological processes (*p* < 0.05) for gene/protein pairs in Quadrants 1, 3, 7, and 9. Table S5. Significantly enriched KEGG pathways (*p* < 0.05) for gene/protein pairs in Quadrants 1, 3, 7, and 9. Table S6. Genes/proteins annotated in the ko01100, ko01110, and ko01200 pathways. Table S7. Gene/protein pairs involved in amino acid biosynthesis and pathways. Table S8. Functional annotation of genes involved in amino acid biosynthesis and metabolism pathways. Table S9. Expression profiles of ABC transcript genes at transcript and/or protein level during vernalization. Table S10. Quantitative analysis of differentially abundant phytohormones across vernalization stages. Table S11 Genes/proteins involved in BR signaling pathway.

## Data Availability

Data that support the findings were derived from the following resources available in the public domain: GSA (CRA010151) and iProX (PXD045106). All data generated or analyzed in this study are included in this published article and its supplementary information files.

## Abbreviations

2HJA: Dihydrojasmonic acid
6DCS: 6-Deoxocastasterone
ABA: Abscisic acid
ACC: 1-Aminocyclopropanecarboxylic acid
BL: Brassinolide
BP: Biological process
BR: Brassinosteriod
CC: Cellular component
CS: Castasterone
cZT: Cis-zeatin
DEG: Differentially expressed gene
DEP: Differentially expressed protein
FPKM: Fragments Per Kilobase of exon model per Million mapped fragments
GA: Gibberellin
GO: Gene Ontology
HPLC–MS/MS: High-performance liquid chromatograph-tandem mass spectrometer
I3CA: Indole-3-carboxaldehyde
IAA: Indole-3-acetic acid
iPA: N6-isopentenyladenine
IPA: Isopentenyl adenosine
JA: ( ±)-Jasmonic acid
KEGG: Kyoto Encyclopedia of Genes and Genomes
KO: KEGG Orthology
KT: Kinetin
MeIAA: Methyl indole-3-acetate
Nr: Non-redundant protein
SA: Salicylic acid
SAM: Shoot apical meristem
SDS-PAGE: Sodium dodecyl sulfate polyacrylamide gel electrophoresis
TCA: Trichloroacetic acid
TF: Transcript factor
tIAA: Trans-indole-3-acrylic acid
TMT: Tandem mass tags
TY: Typhasterol
tZR: Trans-zeatin-riboside
tZT: Trans-zeatin
UHPLC-MRM-MS/MS: Ultra-high performance liquid chromatography-multiple reaction monitoring-tandem mass spectrometry
WGT: Whole-genome triplication
