# Supplementary material for: Integrative multi-omics analyses reveal transcriptional, metabolic, and hormonal regulation of vernalization-induced flowering in Wucai (Brassica rapa)
Source: Mol Hortic. 2026 Oct 10;6:72. doi: 10.1186/s43897-026-00246-7 (PMC13653411; doi:10.1186/s43897-026-00246-7)
Supplement: Supplementary file 1 [file 43897_2026_246_MOESM1_ESM.docx]

**Fig. S1 Quality assessment of RNA-Seq data and statistics of proteomic identifications.**

A, Total number of reads and bases for each sample; B, Average GC content, Q20, and Q30 scores across samples. C, Alignment statistics of sequences to the reference genome. D, Number of spectra, peptides, and proteins identified in the proteomic datasets.

**Fig. S2 Pearson’s correlation coefficients and principal component analyses.**

A and B, Pearson’s correlation coefficients among biological replicates of Wucai shoot apices from the transcriptome (A) and proteome (B). C and D, Principal component analysis (PCA) of replicates from the transcriptome (C) and proteome (D).

**Fig. S3 Concordant tests between proteins and cognate mRNAs.**

A-C, Venn diagrams of transcripts and proteins from V0&V15, V0&V30, and V15&V30 comparisons; the numbers between protein and transcript mean the pairs of expressed mRNA and their coding proteins. D-F, Concordance tests for V0&V15, V0&V30, and V15&V30 comparisons; dots of different colors represent pairs of mRNAs and their coding proteins; Red dots mean fold change (FC) of both transcript and protein levels ≧ 0; cerulean-blue dots mean FC of transcript levels >0 and FC of protein levels < 0; blue dots mean FC of both transcript and protein levels < 0; green dots mean FC of transcript levels <0 and FC of protein levels > 0.

**Fig. S4 Statistics and expression trends of the TFs.**

A, Number of TFs identified among DEGs and DEPs in the V0&V15, V0&30, and V15&V30 comparisons. B-C, Expression trends of TFs based on transcript levels (B) and protein abundance (C).

**Fig. S5 Distribution of transcript/protein pairs across a nine-quadrant plots.**

A, Number of transcripts/proteins pairs in each quadrants. B, Percentage of Q5 and Q1, 3, 7, 9. Q1~Q9, mean Quadrant 1~9.

**Fig. S6 GO enrichment of biological processes for gene/protein pairs in the quadrants 1, 3, 7, and 9.**

A-C, Significantly enriched GO biological processes for V0&V15 (A), V0&V30 (B), and V15&V30 (C) comparisons.

**E**

**Fig. S7 Venn diagrams of enriched KEGG pathways.**

A, Venn diagram showing the overlap of significantly enriched KEGG pathways across V0&V15, V0&V30, and V15&V30 comparisons. B-C, Venn diagrams of gene/protein pairs in the metabolic pathway (ko01100; B) and biosynthesis of secondary metabolites (ko01110; C). D, Venn diagram showing overlap of gene/protein pairs between the ko01100/ko01110 pathways and the carbon metabolism pathway (ko01200) during vernalization. E, Transcript levels of genes involved in sucrose response and sucrose-mediated signaling pathways.

**Fig. S8 Activities of sucrose synthase and sucrose phosphate synthase during vernalization process.**

**Fig. S9 Genes involved in amino acid biosynthesis and metabolism.**

A, Venn diagram showing gene/protein pairs involved in amino acid biosynthesis and amino acid metabolism pathways. B, Heatmap showing the transcript levels and protein abundance of eight key genes involved in the biosynthesis of amino acids and amino acid metabolism pathways; heatmap colors indicate normalized content (log_2_); red indicates high abundance, and blue indicates low abundance.

**Fig. S10 Assay of amino acid metabolites.**

A-C, Volcano plots of differential amino acid metabolites (DAMs) in V0&V15 (A), V0&V30 (B), and V15&V30 (C); points represent metabolites; the x-axis represents the fold change; the y-axis represents the *p*-value of the t-test (logarithm with base 10); the size of the scatter point represents the OPLS-DA VIP (Variable Importance in Projection) value; the blue dots represent down-regulated DAMs, the red dots represent up-regulated DAMs, and the gray dots represent non-significant metabolites; the top 5 DAMs were labeled. D, Heatmap of DAM abundances; row scales were normalized and the values were scaled by log2; heatmap colors indicate count of different amino acid metabolites, and red indicates high abundance, and blue indicates low abundance. E-H, Fold changes of glycine (E), serine (F), proline (G), and arginine (H) between V0 and V15, and V0 and V30. I，Activities of glycine, serine and threonine, arginine and proline synthases.

**Fig. S11** Representative plant phenotypes following treatment with proline.

A, Representative plant phenotypes following treatment with different concentrations of proline; C0.0, C0.5, and C2.0 represent proline concentrations of 0.0, 0.5, and 2.0 mM, respectively; red boxes indicate early bolting after spraying proline at concentrations of 2.0 mM; images were taken approximately110 days after planting; scale *bar* = 10 cm. B, Expression of flowering related genes; error bars indicate the means ± SEs; lowercase letters indicate significant differences between stages (Duncan’ s test, *p* < 0.05).

**Fig. S12 Analysis of ABC transporters and phytohormone abundance.**

A, Venn diagram of ABC transporter gene/protein pairs detected at both mRNA and protein levels. B-D, Volcano plots of phytohormones in V0&V15 (B), V0&V30 (C), and V15&V30 (D), respectively.

**Fig. S13 Expression of BR biosynthesis genes.**
Expression analysis of BR biosynthesis-related genes based on transcriptomic (A) and proteomic (B) data. *BcDET2* was the only gene found to be significantly upregulated at both mRNA and protein levels during vernalization.

**Fig. S14 Functional characterization of *BcDET2* in *Arabidopsi*s.**

A, Phenotypes of the *BcDET2*/*det2* and *BcDET2*/Col-0 lines compared to controls; scale *bar* = 5 cm. B, Relative expression levels of *BcDET2* gene in the transgenic lines. C, Flowering time for the *BcDET2*/*det2* and *BcDET2*/Col-0 lines (*n* = 10). D, Expression analysis of key flowering genes. E, BR concentrations in leaves from *Arabidopsis* Col-0, *det*2, *BcDET2*/*det2*, and *BcDET2*/Col-0 plants; BL, brassinolide; CS, castasterone; 6DCS, 6-deoxocastasterone; TY, typhasterol; error bars indicate the means ± SEs; significant differences compared to the relevant control were determined by Student's t-test (**p* < 0.05, ***p* < 0.01, *** *p* < 0.001); ND, not detectable.
